# High-throughput method for improving rice AGB estimation based on UAV multi-source remote sensing image feature fusion and ensemble learning

**DOI:** 10.3389/fpls.2025.1576212

**Published:** 2025-04-15

**Authors:** Jinpeng Li, Jinxuan Li, Dongxue Zhao, Qiang Cao, Fenghua Yu, Yingli Cao, Shuai Feng, Tongyu Xu

**Affiliations:** ^1^ College of Information and Electrical Engineering, Shenyang Agricultural University, Shenyang, China; ^2^ National Digital Agriculture Sub-center of Innovation (Northeast Region), Shenyang, China; ^3^ Key Laboratory of Intelligent Agriculture in Liaoning Province, Shenyang, China

**Keywords:** rice, aboveground biomass, unmanned aerial vehicle (UAV), multi-source remote sensing images, ensemble learning

## Abstract

**Introduction:**

The rapid and non-destructive estimation of rice aboveground biomass (AGB) is vital for accurate growth assessment and yield prediction. However, vegetation indices (VIs) often suffer from saturation due to high canopy coverage and vertical organs, limiting their accuracy across multiple growth stages. Therefore, this study utilizes UAV-acquired RGB and multi-spectral (MS) images during several critical rice stages to explore the potential of multi-source data fusion for accurately and cost-effectively estimating rice AGB.

**Methods:**

High-frequency texture features were extracted from RGB images using discrete wavelet transform (DWT), while low-order color moments in RGB and Lab color spaces were calculated. VIs were derived from MS images. Feature selection combined statistical analysis and modeling techniques, with collinearity removed through the Variance Inflation Factor (VIF). The relationships between AGB and the selected features were then analyzed using multiple fitting functions. Both single-type and multi-type features were used to develop individual and ensemble machine learning (ML) models for rice AGB estimation.

**Results:**

The findings indicate that: (i) Single-type features result in significant errors and low accuracy within the same sensor, but multi-feature fusion improves performance. (ii) Fusing RGB and MS image features enhances AGB estimation accuracy over single-sensor features. (iii) Ensemble ML models outperform individual models, providing higher accuracy and stability, with the best model achieving an R^2^ of 0.8564 and RMSE of 169.32 g/m^2^.

**Discussion:**

This study demonstrates that multi-source UAV image feature fusion with ensemble learning effectively leverages complementary data strengths, offering an efficient solution for monitoring rice AGB across growth stages.

## 1 Introduction

Rice is one of the most crucial food crops globally, playing a significant role in food security and sustainable development. Above-ground biomass (AGB) is a key agronomic parameter for evaluating crop growth. It serves as an important indicator of overall crop health and productivity, is directly linked to crop yield, and is also critical for monitoring rice growth and managing rice fields effectively (Geng et al., 2021; Shu et al., 2022). Therefore, efficient, non-destructive, and precise monitoring of AGB at the field scale is vital for high-throughput screening in rice breeding. It also holds significant value for decision-making in rice production management and yield forecasting.

Traditionally, the measurement of AGB has been carried out through manual, destructive sampling in the field, a method that can damage the crop and is time-consuming (Yan et al., 2019). Furthermore, manual sampling is restricted to small areas due to the limited number of sampling points, making it difficult to monitor crop AGB over large areas efficiently and quantitatively (Xu et al., 2018; Kumar et al., 2021). Remote sensing, with its efficiency and convenience, has proven to be a valuable tool for obtaining crop AGB at the field scale (Fei et al., 2021). Previous studies have employed various types of remote sensing data from sensors such as multi-spectral (MS) and hyperspectral (HS) sensors mounted on remote sensing platforms (satellites, unmanned aerial vehicles (UAVs), and ground-based platforms) to monitor crop AGB (Xu K. et al., 2019; Xu M. et al., 2019). Ground-based platforms provide high-resolution spectral data with excellent monitoring accuracy, but their limited coverage restricts their application over larger areas. Satellite platforms can offer synchronous observation across extensive regions, but issues such as revisit intervals and atmospheric conditions often hinder the acquisition of high-frequency, high-resolution data at specific regional scales (Zhang et al., 2019; Weiss et al., 2020). Recently, advancements in UAV remote sensing technology, particularly the miniaturization of sensors have allowed UAV platforms to capture high-resolution remote sensing data flexibly, efficiently, and at a relatively low cost. This has addressed the limitations of both ground-based and satellite platforms and has made UAVs an increasingly important tool for modern precision agriculture research (Sagan et al., 2019; Yang et al., 2020).

Usually, vegetation indices (VIs), textures, and structural parameters (e.g., plant height, cover, etc.) extracted from remote sensing images are commonly used as key features for estimating crop AGB (Shu et al., 2021; Luo et al., 2022). Among them, VIs reflect changes in reflection peaks and absorption valleys associated with variations in the physicochemical properties of crop organs (intra-crop features), while texture and structural parameters quantify changes in crop canopy structure (extra-crop features) (Yue et al., 2018). However, research has indicated that relying solely on VIs for estimating crop AGB across multiple growth stages can lead to unstable results. For instance, factors such as soil and water background effects, as well as spectral saturation, can adversely affect the performance of VIs (Zha et al., 2020; Yue et al., 2023). To address these issues, prior studies have sought to integrate features from visible images (e.g., texture, structure) with complementary multi-source data, such as hyperspectral or multi-spectral images (e.g., VIs). For instance (Liu et al., 2023a), investigated the feasibility of using multi-source remote sensing feature fusion to estimate potato AGB across multiple growth stages. They extracted optical vegetation indices, texture, and structural features from UAV-captured RGB and hyperspectral images, and combined Gaussian process regression (GPR) with partial least squares regression (PLSR). Their results demonstrated that multi-source remote sensing data fusion could effectively estimate AGB at different growth stages of potatoes, with the GPR method showing superior estimation accuracy. Similarly (Cen et al., 2019), used a UAV with a dual-frame camera to capture RGB and MS images of rice canopies, extracting plant height and VIs. By combining these features in a random forest (RF) regression model, they successfully monitored dynamic changes in rice AGB under different nitrogen treatments. These studies highlight that image features such as rich color information, high-frequency textures, and structural features extracted from RGB images can compensate for the limitations of spectral vegetation indices in estimating crop AGB. The fusion of multi-source remote sensing data has gained significant attention for AGB estimation across multiple growth stages of crops, and its full potential for application remains to be further explored.

UAV-based remote sensing platforms offer the advantage of capturing large amounts of high-dimensional data, which, however, presents challenges for modeling (Montesinos-López et al., 2017). Recent advancements in computer science have led to the widespread adoption of machine learning (ML) algorithms in quantitative remote sensing research (Shu et al., 2021). Algorithms such as random forest (RF), extreme learning machine (ELM), and support vector machine (SVM) have been increasingly used to develop models for estimating crop phenotypic traits (Fu et al., 2019; Ji et al., 2022). These ML algorithms have improved the accuracy and stability of crop trait estimations using UAV remote sensing data. However, using a single ML algorithm, especially with limited training data, can lead to overfitting (Masoud et al., 2023). To address this issue and enhance model performance, various solutions have been proposed (Ribeiro and Coelho, 2020). One of the most effective is ensemble learning, which integrates the predictions from multiple ML models to achieve higher accuracy and stability (Yoosefzadeh-Najafabadi et al., 2021). Several studies have shown that ensemble learning outperforms individual machine learning models in estimating crop biomass, yield, and other agronomic traits, offering improved accuracy and robustness. For instance (Ji et al., 2023), applied stacking ensemble learning to estimate the AGB and yield of beans, significantly improving estimation accuracy compared to individual models. Similarly (Fei et al., 2023), utilized multi-sensor data fusion and stacking ensemble learning to improve wheat yield predictions. Ensemble learning methods typically include bagging (Zhang et al., 2021), boosting (Zhang et al., 2020), and stacking (Tao et al., 2023). Among ensemble learning methods, bagging and boosting are typically limited to integrating models of the same type, such as decision trees, which makes it challenging to leverage the strengths of different model types. In contrast, stacking is a hierarchical model integration approach that allows for the use of diverse base learners, each trained on the dataset. The outputs of these base learners are combined into a new training set, which is then used as input for a meta-learner to generate the final decision (Healey et al., 2018). By aggregating the predictions from multiple base learners, the stacking ensemble method can significantly enhance the accuracy, robustness, and generalization capabilities of the estimation model (Ju et al., 2018; Feng et al., 2020).

In rice biomass research, single machine-learning models are commonly applied to estimate AGB using remote sensing data. However, there is a limited exploration of combining multi-source image features with ensemble learning techniques to estimate rice AGB across different growth stages. Thus, the primary aim of this study is to investigate the potential of using UAV-acquired RGB and multispectral (MS) images for estimating rice AGB at different growth stages by integrating image features (such as high-frequency texture and color features) with spectral features through a stacking ensemble learning method. Specifically, the study has three main objectives: (i) to evaluate the effectiveness of spectral features, color features, and high-frequency texture in estimating rice AGB; (ii) to compare the performance of individual ML models and ensemble learning for rice AGB estimation; and (iii) to assess whether the fusion of multi-source UAV image features and ensemble learning can improve the accuracy and stability of AGB estimation in rice.

## 2 Materials and methods

**Figure 1:**
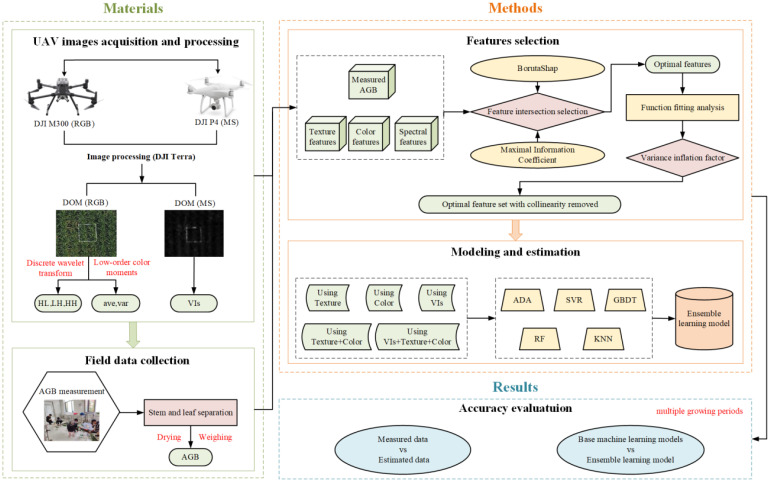
The technical flow of this study.

The workflow of the technical aspects of this study is outlined in **Figure 1**. The primary elements of the research include the acquisition and processing of UAV-based RGB and MS images; the statistical analysis and feature selection of both the image data and biomass measurements; the construction of an ensemble learning model for estimating rice AGB based on multi-source data; and the evaluation of the mode performance in AGB estimation.

### 2.1 Experiment location and design

**Figure 2:**
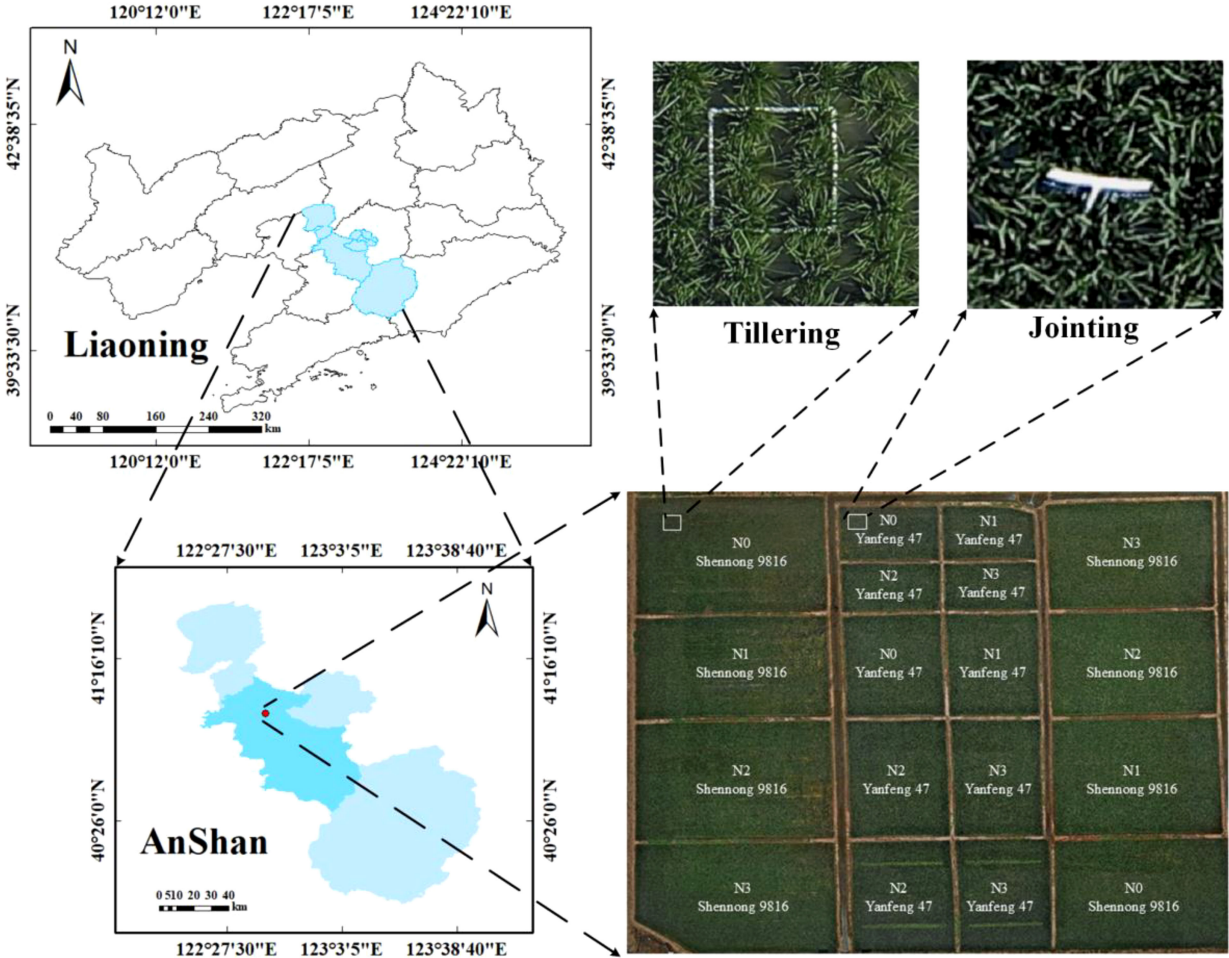
Geographic location of the experimental field and experimental design.

The field experiment was carried out from June to August 2024 at the Precision Agriculture Aviation Research Base of Shenyang Agricultural University located in Haicheng City, Anshan, Liaoning Province, China (**Figure 2**, 122°39′18″E, 40°58′58″N). Haicheng City has a temperate continental monsoon climate, with an average summer temperature of 20°C to 28°C and an average annual precipitation of 721.3 mm. The region features flat terrain, fertile soil, and relatively abundant groundwater resources, which are suitable for rice growth. The experiment comprised 18 plots. According to the principle of five-point sampling in each plot, a total of 90 study areas were marked as regions of interest (ROIs) for data extraction, using 0.5*0.5 m white plastic frames. To assist in identifying the research areas from UAV, a 1.5 m long white signage was inserted in the upper right corner of the white plastic frame, as the canopy was closed from the jointing stage of rice and shaded the white frame. The signage was placed at a distance of one hole of rice. In this study, two rice cultivars (Shennong 9816 and Yanfeng 47) and four nitrogen levels (N0 = 0 kg/hm^2^, N1 = 100 kg/hm^2^, N2 = 200 kg/hm^2^, N3 = 300 kg/hm^2^) were used for the experiment. The rice seedlings of both varieties were transplanted on May 28, 2024. The application rates of phosphate and potassium fertilizers for each plot were determined to be 144 kg/hm^2^ and 192 kg/hm^2^, respectively, following the recommended local dosage. All other field management measures were maintained consistently with local farmland management.

### 2.2 AGB measurements

Ground destructive sampling was carried out on June 27 (tillering), July 22 (jointing), and August 19 (heading) in 2024, respectively. Following the UAV flight operation, three representative plant holes were collected from the uniformly growing area near each of the five white sample squares within each experimental field. These samples were placed in labeled plastic bags and brought back to the laboratory. Subsequently, the plants were rinsed with water. The roots were cut, and the stems and leaves were separated using scissors and placed into envelopes labeled with the corresponding sampling areas. All envelopes containing samples were then placed in an oven and dried at 105°C for 30 min to deactivate the enzyme, followed by further drying at 80°C until constant weight. An electronic scale with an accuracy of 0.01 g was used to peel weigh, and convert to AGB per unit area (g/m^2^) according to the Equation 1.


(1) $$AGB=\frac{m\times n}{3\times s}$$

Where *m* is the dry weight of the rice sample, *n* is the number of rice plants in the sample area (6 cavities in this study), and *s* is the area of the sample (0.25 m^2^).

A total of 270 AGB samples (90 samples in each period) were collected in the three periods, and the biomass statistics of each period are shown in **Table 1**. The statistical indicators included maximum value, minimum value, mean value, standard deviation, and coefficient of variation. Among them, the coefficient of variation of the total AGB dataset of the three growth stages was 48.55%, which illustrated the large influence of the growth stage on the canopy structure of rice.

**Table 1:** Data statistics of AGB.

Parameters	AGB(g/m^2^)			
	All data	Tillering	Jointing	Heading
No. of samples	270	90	90	90
Min	344.64	344.64	548.16	628.09
Max	1868.16	618.72	1290.96	1868.16
Mean	1000.64	451.96	994.65	1343.87
SD	485.81	65.02	195.23	346.70
CV(%)	48.55	14.39	19.63	15.13

### 2.3 UAV image acquisition and processing

UAV image data were collected simultaneously with field sampling. To ensure consistent light and sun angles, all flight missions took place between 11:00 am and 12:00 pm (Beijing time) under windless and cloudless conditions. The MS and RGB images were acquired using P4M and M300 UAV (DJI, Shenzhen, China), respectively. The UAV flew independently on a predefined route during each growth period and maintained the same route plan for image data collection. In this study, the P4M weighing approximately 1487 g, was equipped with six 1/2.9-inch CMOS sensors, including five monochrome sensors for B, G, R, red edge (RE), and near-infrared (NIR), along with an RGB camera. Each sensor had a resolution of 2.08 megapixels and a focal length of 5.74 mm. The UAV was equipped with a sun sensor positioned on top to automatically adjust the reflectivity according to the intensity of sunlight, ensuring the consistency of data under varying weather conditions. The M300 had a maximum payload of 2.7 kg, a maximum endurance of approximately 55 minutes, and was equipped with a Zenmuse P1 (DJI, Shenzhen, China) visible light camera. The camera weighed about 800 g and has 45 million effective pixels with a maximum resolution of 8192 × 5460. Flight operations were carried out at an elevation of 30 m and featured an overlap ratio of 80%.

After the completion of the flight operation, Terra (DJI, Shenzhen, China) software was applied to correct and stitch the UAV RGB and MS images. The UAV RGB and MS image files acquired in each period were imported into the software, which automatically read the positioning and attitude system data, along with configuration details. For MS image stitching, it was necessary to perform reflectance correction using the standard whiteboard image captured by the UAV before takeoff to generate reflectance images. Subsequently, the processed RGB and MS images were saved as GeoTIFF files with ground sampling distance (GSD) of 0.36 and 1.86 cm/pixel, respectively. In both images, ENVI 5.3 software was employed to define the regions of interest (ROIs) according to the areas marked by the white plastic frames. The “Subset Data from ROIs” was then utilized to crop the study area and saved as a GeoTIFF file for further data extraction.

### 2.4 Feature extraction

Compared with MS images, RGB images acquired at the same flight altitude have higher spatial resolution. Therefore, in this study, RGB images are used to extract image feature information such as texture and color, and MS images are used to obtain spectral feature information.

#### 2.4.1 VIs calculation

An effective approach commonly used in crop growth monitoring studies is to construct Vegetation Indices (VIs) with specific bands to assess the status of crop growth. These VIs have specific physical meanings, which enhance the specific characteristics of the vegetation and reduce the impact of factors like solar irradiance, soil and water background, etc (Yue et al., 2018). According to previous studies on estimating crop AGB using VIs (Cen et al., 2019; Yang et al., 2022), We selected five VIs calculated based on MS images with specific names and definitions shown in **Supplementary Table S1**. The band calculation tool (Band Math) in ENVI 5.3 software was utilized to carry out band operations and compute these VIs. Subsequently, the average value of each VI within each designated region at different periods was extracted as the final VIs of the study area.

#### 2.4.2 Discrete wavelet transform analysis

**Figure 3:**
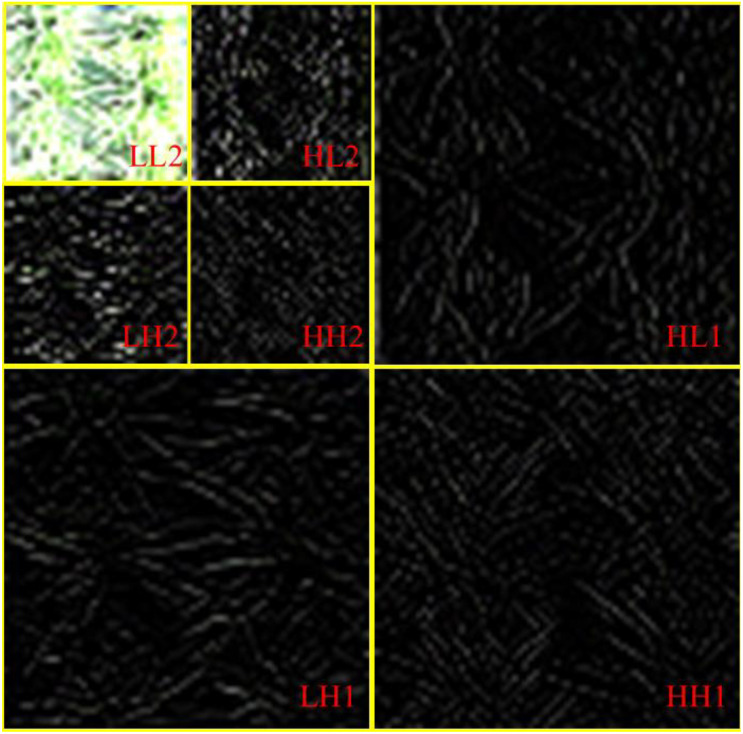
Discrete wavelet decomposition process of RGB images.

In recent years, discrete wavelet transform (DWT) technology with multi-resolution and time-frequency localization characteristics has gradually become an effective tool for texture analysis and has been applied in crop phenotypic trait estimation studies (Zhou et al., 2022). DWT decomposes signals layer by layer through a set of high-pass (H) and low-pass (L) filters, effectively extracting contour features and detailed information at different scales (Yue et al., 2019). Each decomposition produces three high-frequency sub-images in horizontal (LH), vertical (HL), and diagonal (HH) directions, as well as a low-frequency sub-image (LL) containing main contours. Iterative decomposition of the low-frequency sub-image can generate finer-scale sub-images. In this study, the two-level DWT decomposition process for rice UAV imagery is illustrated in **Figure 3**. As the decomposition level increases, the spatial resolution of sub-images decreases. Since high-frequency details are generally considered to represent dense crop canopies (Yue et al., 2021), this study selected a single-level DWT decomposition for RGB images to prevent loss of image details. For DWT implementation, five commonly used wavelet basis functions were chosen, including four orthogonal bases haar, daubechies3 (db3), symlets6 (sym6), and coiflet3 (coif3), and one non-orthogonal basis biorthogonal 3.3 (bior3.3) from the MATLAB wavelet toolbox. To simplify the representation of decomposition results, each wavelet basis function was paired with the high-frequency components in different directions. For example, “haar_HH” denotes the diagonal high-frequency information obtained using the haar wavelet basis function.

#### 2.4.3 Color moment analysis

Color moments are a simple yet effective method for representing color features, primarily utilizing low-order moments such as the first-order and second-order moments to characterize image color distributions (Ge et al., 2021a). The RGB color space offers intuitive application and widespread adoption, enabling efficient capture of color information, yet exhibits sensitivity to illumination variations that may compromise feature stability (Huang et al., 2020). In contrast, the Lab color space demonstrates perceptual uniformity and illumination invariance, effectively mitigating color feature deviations caused by lighting changes (Larijani et al., 2019). In this study, we computed two low-order color moments for each of the three channels in both RGB and Lab color spaces through the mathematical formulations presented in Equation 2, Equation 3, yielding a total of 12 color features. For simplified representation, we adopt a combined notation of color channels and moment types, for example, R_ave and R_var respectively denote the first-order moment and second-order moment calculated from the R component in RGB space.


(2) $$\mu_{i}=\frac{1}{N}\sum_{j=1}^{N}p_{i,j}$$

(3) $$\sigma_{i}={\left(\frac{1}{N}\sum_{j=1}^{N}{\left(p_{i,j}-\mu_{i}\right)}^{2}\right)}^{\frac{1}{2}}$$

Where *p_i,j_* represents the probability of the pixels of the gray value with *j* in the *i_t_*_h_ color component (*i* = *R*, *G*, *B*, *L*, *a*, *b*), *N* is the total number of pixels in each image, *μ_i_*, *σ_i_* and *ω_i_* represents the first-order moments and second-order moments in each color component, respectively.

### 2.5 Feature selection methods

The Maximal Information Coefficient (MIC), as a non-parametric statistical method, can effectively quantify the strength of linear or nonlinear associations between two variables. With strong generalization capabilities and robustness, it is commonly used for feature selection in machine learning. BorutaShap is a model-based feature selection method that integrates Boruta’s feature competition framework with SHAP visual analysis to achieve stable and interpretable feature screening. Specifically, the Boruta method generates shadow features to compete with original features and evaluates feature importance based on a random forest model. SHAP values quantify the marginal contributions of features to model outputs, providing interpretability of feature importance at both global and local levels. In this study, these two methods are comprehensively utilized to fully explore the statistical relevance of feature variables and the contribution of model decision-making and to improve the comprehensiveness and reliability of feature selection, to screen the feature variables that are highly correlated with the AGB, and to provide more representative input variables for the construction of the subsequent AGB estimation model.

### 2.6 Construction of ensemble model

**Figure 4:**
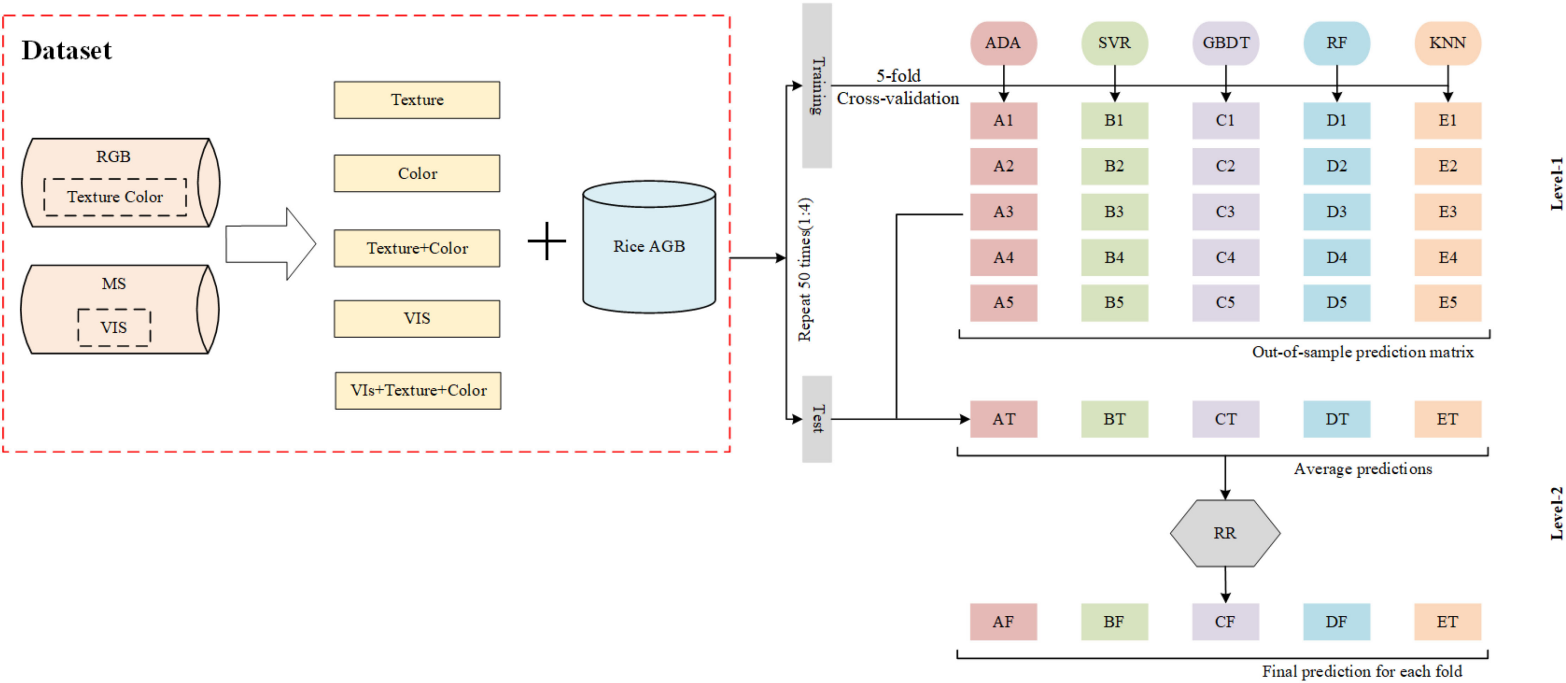
The flow of ensemble learning for estimating rice AGB.

Ensemble learning combines multiple base machine learning models, synthesizing the performance of each model. Compared with a single machine learning model, it can effectively improve the accuracy of regression or classification problems (Li et al., 2020). Stacking regression is a common hierarchical ensemble learning strategy, which is generally divided into two levels: base model and meta-model. The base model includes several different machine learning models that are trained based on the original input data, and the results obtained from the base model are aggregated into a new feature set and applied as new inputs to the meta-model for training to obtain the final results. In this study, the base models include AdaBoost, support vector regression (SVR), gradient boosting decision tree (GBDT), random forest (RF), and K-Nearest Neighbor (KNN), and the meta-model used ridge regression (RR). The range of parameter settings for each model is shown in **Supplementary Table S9** of the Supplementary Materials, and the parameters of the individual models were tuned using the grid search cross-validation algorithm to improve the performance of the ML models. In this study, the original data were split into training and test sets in the ratio of 4:1 and repeated 50 times to eliminate random errors. The same data partitioning method was applied across all data sources to ensure a fair comparison of the estimation accuracies of different models. To prevent overfitting, five-fold cross-validation was employed during the training of each model. The framework for the ensemble learning model is illustrated in **Figure 4**.

### 2.7 Model performance evaluation

In this study, the dataset was divided into training and validation sets according to 1:4, and the model results were averaged over 50 times to assess the stability and accuracy of the estimates. The accuracy of rice AGB estimation was quantitatively assessed using the coefficient of determination (R²) and root mean square error (RMSE), calculated according to Equations 4, 5.


(4) $$R^{2}=1-\frac{\sum_{i=1}^{n}{\left({\hat{y}}_{i}-y_{i}\right)}^{2}}{\sum_{i=1}^{n}{\left(y_{i}-\bar{y}\right)}^{2}}$$

(5) $$RMSE=\sqrt{\frac{\sum_{i=1}^{n}{\left({\hat{y}}_{i}-y_{i}\right)}^{2}}{n}}$$

Where *y_i_* and ${\hat{y}}_{i}$ are the measured and estimated AGB of rice, respectively, $\bar{y}$ are the mean values of AGB of rice, and *n* is the total number of samples. A higher *R^2^* and lower *RMSE* correspond to a higher accuracy of AGB estimation in rice.

## 3 Results and analysis

### 3.1 Statistical analyses of biomass and characterization variables

To assess the reliability of aboveground biomass (AGB) and characteristic variable data in this study and ensure their validity for constructing AGB estimation models, the Shapiro-Francia test was employed to conduct normality tests on AGB and characteristic variables (**Supplementary Table S2**). Results revealed non-normal distributions for all datasets across growth stages (p<0.05). Consequently, the Kruskal-Wallis test was applied to analyze distribution differences of AGB and characteristic variables among distinct growth stages (**Supplementary Table S3**), with the Dunn post-test further evaluating significant inter-stage differences (**Supplementary Table S4**). The Kruskal-Wallis test demonstrated significant AGB variations across growth stages (p<0.05), consistent with rice growth patterns. All characteristic variables also exhibited statistically significant distributional differences corresponding to AGB dynamics, indicating their potential value for AGB estimation. Dunn post-test specifically identified significant AGB differences between tillering-jointing, jointing-heading, and tillering-heading stages (p<0.05), with particularly pronounced disparities between tillering-heading stages, aligning with rice phenological characteristics. Regarding characteristic variables, VIs showed significant differences between tillering-jointing and tillering-heading stages (p<0.05) but not between jointing-heading stages (p>0.05), suggesting possible VIs saturation during jointing-heading stages that may compromise multi-stage AGB estimation. High-frequency texture features exhibited significance across all growth stage combinations (p<0.05), indicating that it can effectively reflect the changes in different fertility stages of rice and is valuable for AGB monitoring. In the results of color moment analysis, the test results of the tillering-heading stage were all significant (p<0.05), which was in line with the trend of rice color characteristics changes in the two growth stages. However, some of the color moment changes were not significant at the tillering-jointing and jointing-heading stage, for example, L_ave (p>0.05) at the tillering-jointing stage and R_ave (p>0.05) at the jointing-heading stage, implying that some of the color moments change slowly and may have a limited contribution to the AGB estimate. The results of the above statistical analyses suggest that the features extracted in this study can be used to monitor changes in rice AGB. However, it may be difficult for a single feature variable to reflect the complexity of AGB changes, so the selection and integration of feature variables, especially those that show significant changes across growth stages, will help construct a more accurate model for AGB estimation.

### 3.2 Feature selection analysis based on the combination of statistics and modeling

**Figure 5:**
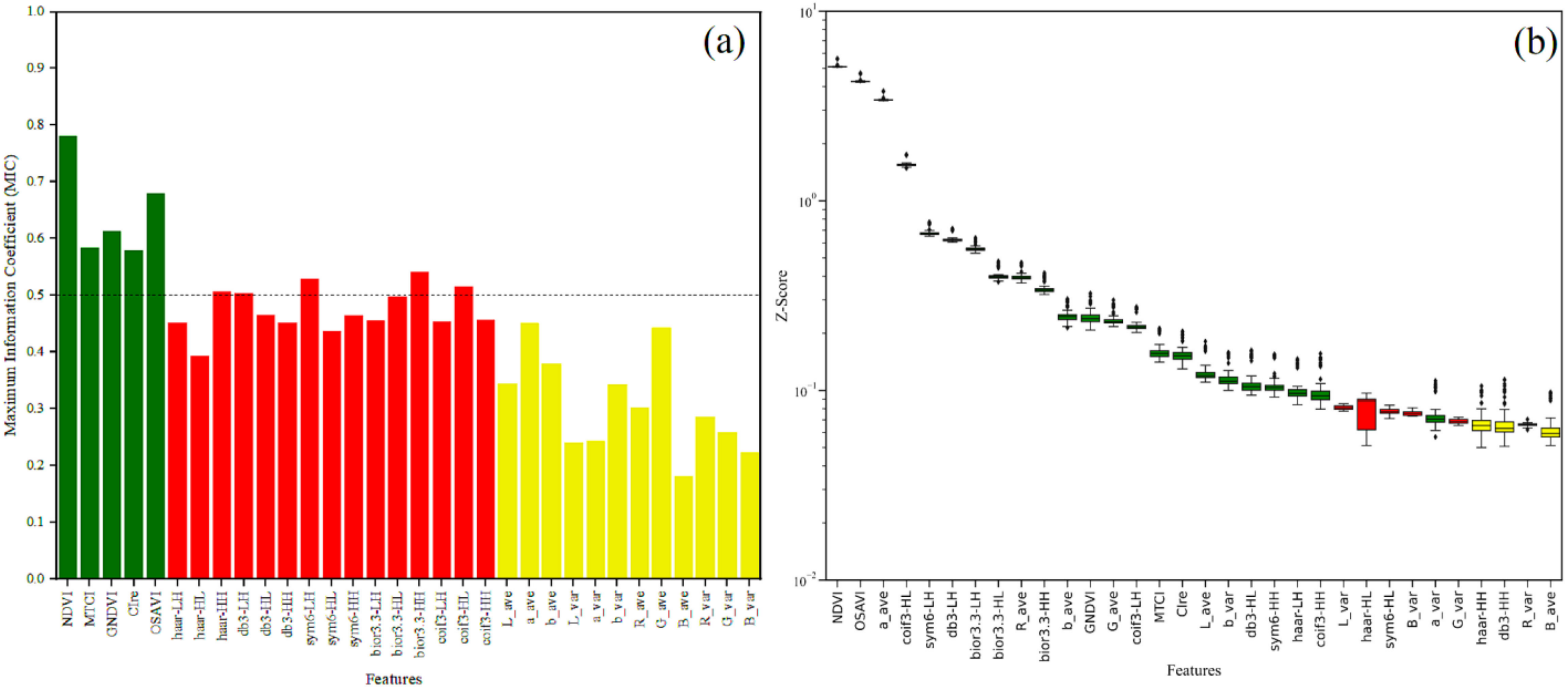
Results of feature selection based on MIC and BorutaShap. **(a)** MIC, **(b)** BorutaShap.

The results of combining MIC with BorutaShap for feature screening are shown in **Figure 5**. Specifically, **Figure 5a** shows the MIC values of VIs, high-frequency texture and color moment features for AGB. Compared to high-frequency texture and color moments, there are higher MIC values between VIs and AGB, indicating a stronger correlation between VIs and AGB. Based on related studies (Cao et al., 2020) and the results of current research, a total of 10 features with stronger correlation with AGB, namely NDVI, MTCI, GNDVI, CIre, OSAVI, haar-HH, db3-LH, sym6-LH, bior3.3-HH and coif3-HL, were screened using MIC=0.5 as the threshold value. The results of selecting features based on the BorutaShap model are shown in **Figure 5b**, where the importance of each feature in the model is distinguished by different colors, respectively. Among them, green means important features, red means unimportant features, and yellow means pending features (uncertain importance). Based on the statistics of the results of 100 tests, the BorutaShap model selected a total of 23 important features, 6 unimportant features, and 3 pending features. To further obtain the final features, this study combined the results of the two methods and prioritized the intersection of the features obtained by the two methods, and a total of 9 features were preferred, including NDVI, MTCI, GNDVI, CIre, OSAVI, db3-LH, sym6-LH, bior3.3-HH, and coif3-HL. To obtain the important features more comprehensively, for the remaining important features and pending features selected by the BorutaShap model, this study preferred the features extracted from the same wavelet basis function and the same color space based on the maximum value of MIC and selected a total of four features, including haar-HH, a_ave, b_var, and G_ave. In summary, this study combined the two feature screening methods to finally select 13 important features.

### 3.3 Evaluation of the relationship between features and AGB

#### 3.3.1 Relationship analysis between VIs and AGB

**Figure 6:**
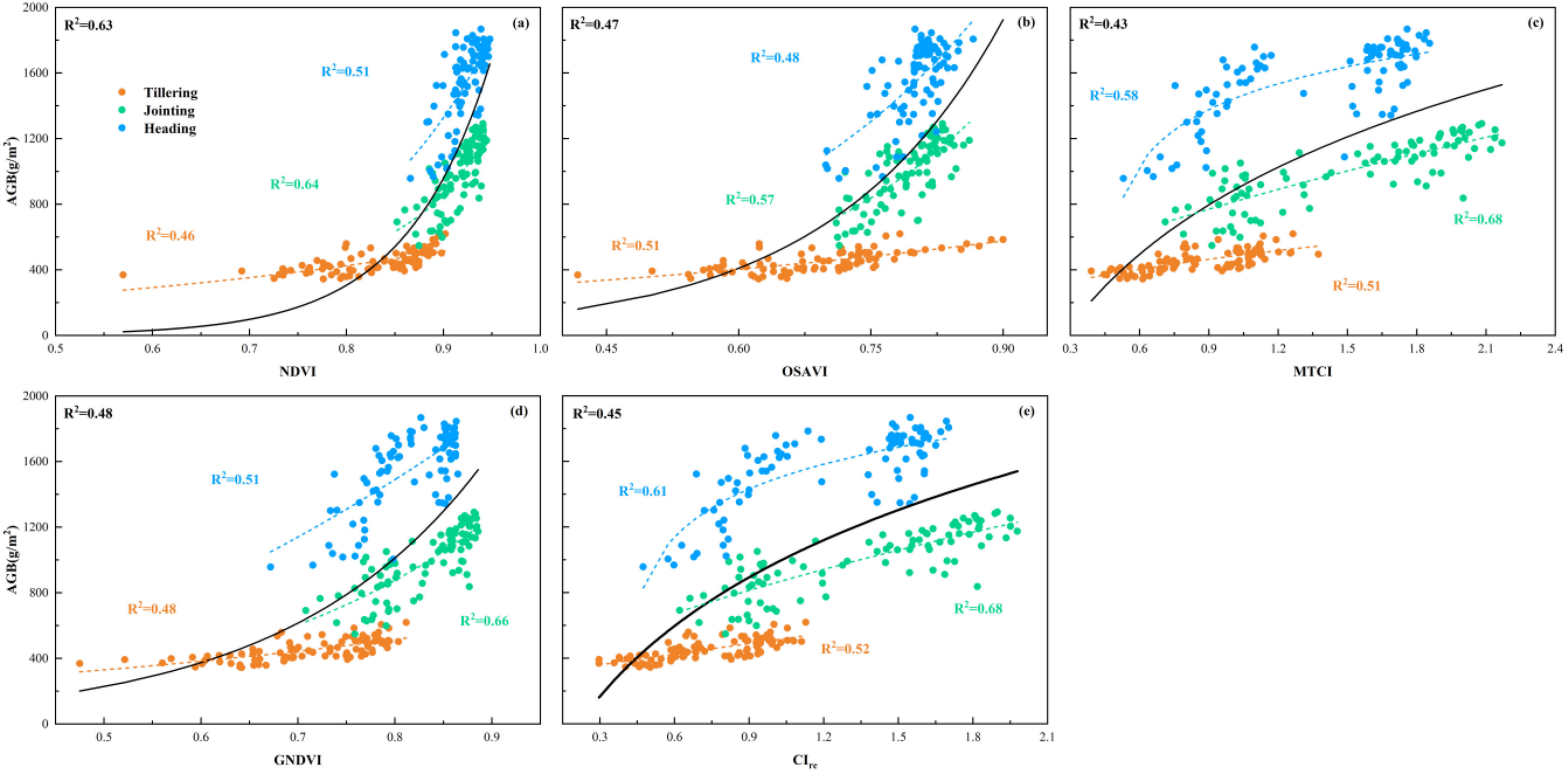
Relationship between VIs and AGB. **(a)** NDVI, **(b)** OSAVI, **(c)** MTCI, **(d)** GNDVI, **(e)** CI_re_.

To assess the relationship between the optimal features and AGB, this study used linear, exponential, logarithmic, and power functions to establish the estimation model of AGB and determine the relationship between the features and AGB. The fitting results of all features and AGB are shown in **Supplementary Tables S5**–**S8**. **Figure 6** presents the optimal relationships between VIs and rice AGB. The VIs account for approximately 43-63% of the observed variation in rice AGB across multiple growth stages, where MTCI and CIre exhibited a logarithmic relationship with AGB, while GNDVI, NDVI, and OSAVI demonstrated an exponential relationship. Notably, NDVI showed the most accurate estimation of AGB at multiple growth stages, achieving an R² of 0.63. The results in **Figure 6** also highlight that the performance of the same VIs fluctuated considerably among the different stages of rice growth. Specifically, the nonlinear relationship between VIs and rice AGB followed an increasing and then decreasing trend as the growth stage progressed. As shown in **Figure 6a**, the relationship between NDVI and AGB showed that tillering (R^2^ = 0.46) was weaker than jointing (R^2^ = 0.64), and the jointing was stronger than heading (R^2^ = 0.51). In the single growth stage of rice, the highest nonlinear relationship between VIs and rice AGB was found at the jointing stage. Among them, two VIs based on the red-edge (MTCI: R2 = 0.68 and CIre: R2 = 0.68) had the best estimation performance at the jointing stage. At the tillering stage, CIre (R2 = 0.52) and MTCI (R2 = 0.51) showed consistent results with those at the jointing stage, but the overall estimation performance was significantly lower compared to the jointing stage. However, the performance of estimating AGB among all VIs at the heading stage showed an overall decreasing trend compared to the jointing stage. In addition, the performance of VIs was generally stronger at the individual rice growth stage than at multiple growth stages, suggesting a limitation in the application of VIs for estimating AGB at multiple growth stages in rice.

#### 3.3.2 Relationship analysis between image features and AGB

**Figure 7:**
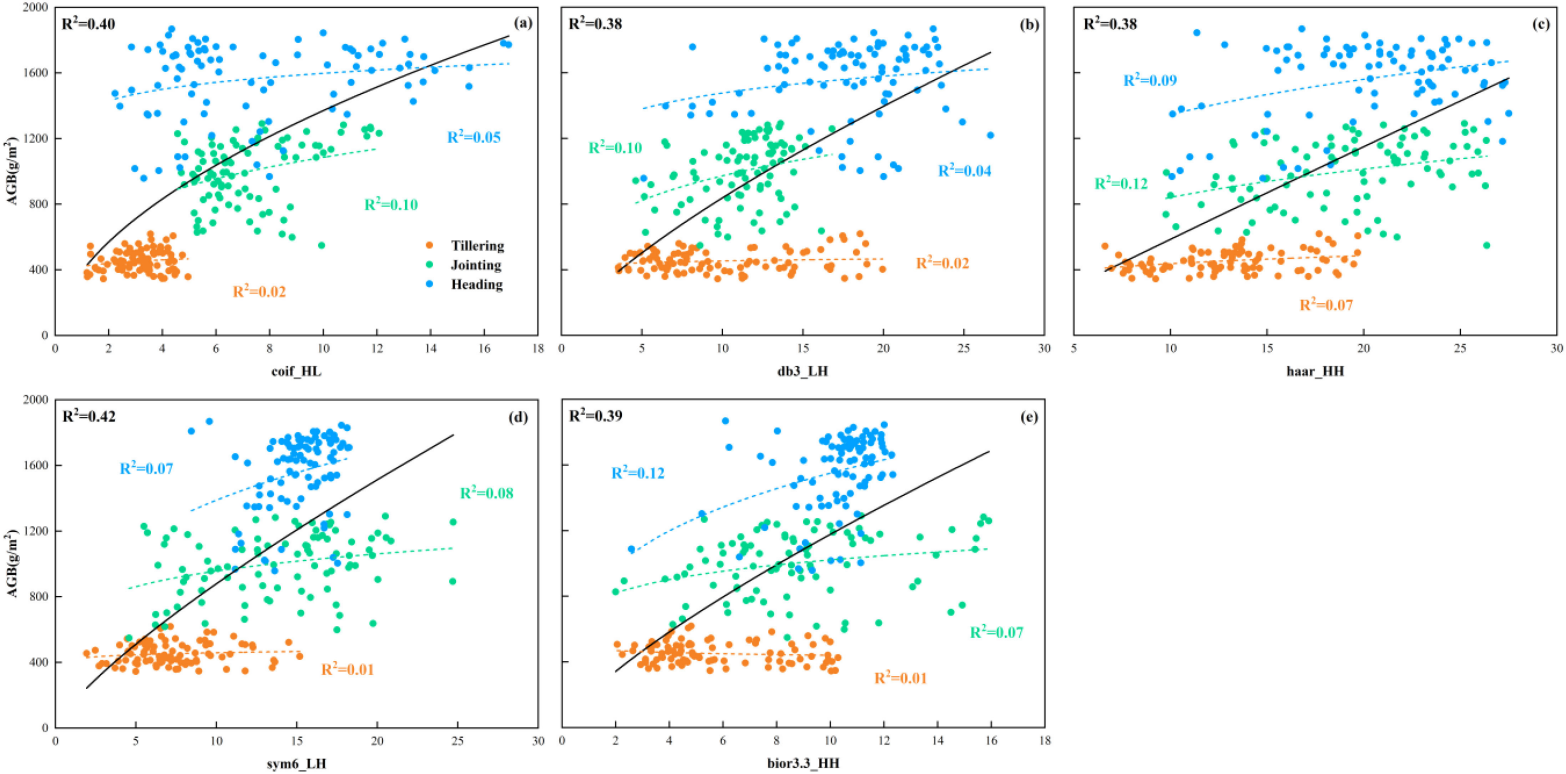
Relationship between high-frequency texture and AGB obtained based on different wavelet basis functions. **(a)** coif3_HL, **(b)** db3_LH, **(c)** haar_HH, **(d)** sym6_LH, **(e)** bior3.3_HH.

**Figure 7** shows the best relationship between the optimal high-frequency texture features extracted using various wavelet basis functions and rice AGB. The optimal power function relationship between the selected high-frequency texture features and rice AGB across multiple growth stages explains 38-42% of the variation in rice AGB, highlighting the effectiveness of high-frequency texture features in capturing key information about rice growth. Among the three individual growth stages, the accuracy of estimating AGB at single growth stages using high-frequency texture was overall low, while the accuracy of estimating AGB at the jointing stage was generally better than that at the tillering and heading stages. This may be because the rapid growth of leaves at the jointing stage has a more significant effect on AGB, and high-frequency texture can effectively reflect this growth change. In contrast, the selected high-frequency textures extracted using different wavelet basis functions provided significantly more accurate AGB estimates across multiple growth stages compared to estimates from a single growth stage. The results demonstrate that high-frequency texture features extracted from RGB images using wavelet transform effectively capture the changes in rice AGB across multiple growth stages, thereby enhancing the accuracy of AGB estimation throughout the rice growth stage. In addition, as shown in **Figure 7**, the difference in R^2^ variation of rice AGB estimated based on high-frequency textures preferred by different wavelet basis functions is small in rice multi-growth and single-growth stages. This suggests that the selection of wavelet basis functions may have less influence on the estimation accuracy of AGB. Therefore, the high-frequency texture of RGB images extracted using the wavelet transform technique can be used for the estimation of AGB in rice at multiple growth stages.

**Figure 8:**
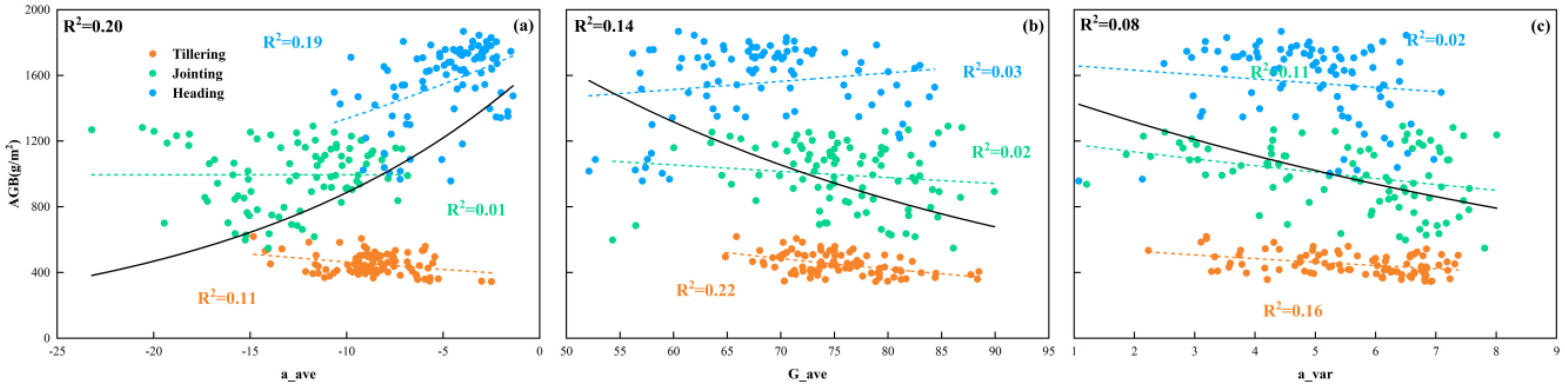
Relationship between different color moments and AGB. **(a)** a_ave, **(b)** G_ave, **(c)** a_var.

The best relationship between the optimal color moments and AGB is shown in **Figure 8**, where the three color moments exhibit an exponential relationship with AGB and explain 8-20% of the variation of rice AGB at multiple growth stages. The poor and fluctuating accuracy of the three color moments in estimating AGB at different growth stages illustrates the unstable ability of color moments to estimate AGB at different growth stages. Even at the multiple growth stages, the accuracy of estimating AGB based on the three color moments was low, with the highest AGB estimation accuracy (R^2^ = 0.20) achieved using a_ave, which also further illustrates the limited ability of color moments to explain AGB compared to VIs and high-frequency texture. However, it is worth noting that due to the limited ability to fit linear and simple nonlinear functions, the complex relationship between color moments and AGB may not be adequately captured, which in turn affects the accuracy of color moments in estimating AGB. Therefore, complex models need to be used to further explore the relationship between color moments and AGB to improve the accuracy of AGB estimation. Additionally, the comparison between the results of a_ave and G_ave might indicate that the a-component in the Lab color space can better reflect the color changes during rice growth while minimizing the impact of environmental factors such as lighting. Thus improving the accuracy in estimating AGB across multiple growth stages. In contrast, the non-independence and light sensitivity of the RGB space cause G_ave to be seriously affected by factors such as light, which leads to its weaker estimation ability for AGB at multiple growth stages. This also suggests that using Lab color space can provide relatively more stable color information in crop growth phenotyping studies compared to RGB color space.

### 3.4 Model performance for estimating rice AGB

**Figure 9:**
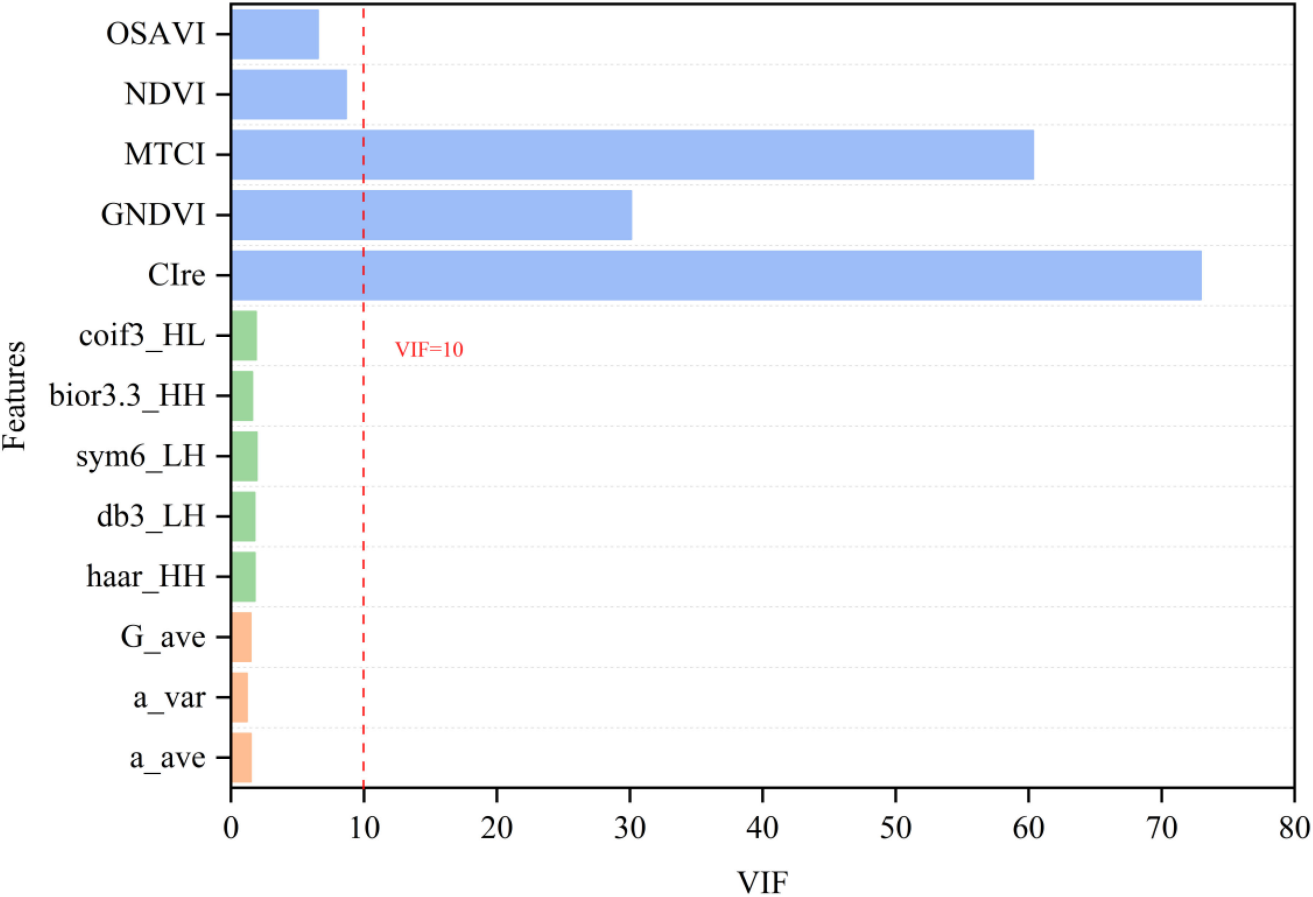
Calculation of variance inflation factors among the characteristic variables.

In this study, a comprehensive dataset containing a total of 13 features, including spectrum, color, and high-frequency texture, was constructed by feature selection. To reduce the impact of multicollinearity on model stability and accuracy, a variance inflation factor (VIF) was used to screen out high-collinearity features. In general, if the VIF value of a feature variable is greater than 10, it means that the feature has high covariance with other features, which may affect the interpretation and prediction ability of the model and needs to be eliminated (Wang et al., 2022). Therefore, 10 feature variables were finally screened out for constructing the AGB estimation model in this study, and the VIF results are shown in **Figure 9**.

To assess the impact of feature fusion and variable covariance on model performance, this study first compares the performance of different machine learning models for estimating the AGB of rice across multiple stages based on a Non-collinearity dataset. The estimation accuracies of each machine learning on the training and validation sets are shown in **Tables 2** and **3**, respectively. The results show that multi-source data fusion significantly improves the estimation accuracy of AGB, and the models show strong generalization ability on both the training and validation sets. Among the RGB extracted features, the model combining texture and color features has higher AGB estimation accuracy than the single-feature model, indicating that multi-feature fusion of a single sensor can effectively improve the model performance. When integrating RGB-derived features with MS spectral data, the AGB estimation accuracy of all ML models improved significantly compared to using single-sensor data alone, further validating the complementary of multi-source sensor data and its enhancement of model accuracy and generalization. Compared to the five base learning models, the ensemble learning model constructed by integrating the five base models using ridge regression (RR) as a meta-model outperforms individual machine learning models, both for multi-type feature data and multi-source image feature data fusion. With dual-sensor data fusion, the ensemble learning model achieved the highest estimation accuracy in both the training and validation sets, with an average R² value of 0.9156 and an average RMSE value of 127.02 g/m² in the training set, and an average R² value of 0.8645 and an average RMSE value of 167.82 g/m² in the validation set. These results indicate that the ensemble learning model effectively combines the advantages of the base model and further improves the accuracy and generalization ability of the model. For the collinearity data (MS dataset and RGB+MS dataset), the models have significantly higher accuracy on the training set than on the non-collinearity dataset. However, the results of the validation set show that the overall difference in the accuracy of the models on the collinearity and non-collinearity datasets is not significant. It is also worth noting that there is a large difference between the training and validation set accuracies of the models in the collinearity dataset. This indicates that the model’s strong dependence on collinearity features may have led to an overfitting of the training set and an inability to generalize effectively to the validation set. It also suggests that the elimination of collinearity needs to be considered in data preprocessing or feature selection to improve the stability and generalization of the model.

**Table 2:** Performance of basic and ensemble learning models for estimating AGB on the training set.

Sensor	Feature type	Variables num	Metrics	Stacking	KNN	GBDT	RF	ADA	SVR
RGB	Tex	5	R^2^	0.7107	0.6905	0.6755	0.6514	0.6859	0.6760
			RMSE(g/m^2^)	254.21	260.43	268.32	276.04	262.32	267.91
	Col	3	R^2^	0.6098	0.5846	0.5635	0.5410	0.5886	0.4719
			RMSE(g/m^2^)	303.15	309.27	329.29	336.31	307.20	353.31
	Tex+Col	8	R^2^	0.8335	0.7928	0.8110	0.8058	0.7891	0.8067
			RMSE(g/m^2^)	184.50	201.94	190.14	196.33	204.91	195.65
MS	Spe (Non-co)	2	R^2^	0.7496	0.6318	0.7010	0.7237	0.6928	0.6401
			RMSE(g/m^2^)	242.01	285.48	256.65	249.55	259.53	280.18
	Spe (Co)	5	R^2^	0.8886	0.8340	0.8328	0.7395	0.8702	0.6777
			RMSE(g/m^2^)	160.77	196.53	197.01	246.23	173.175	273.73
RGB+MS	Spe+Tex+Col (Non-co)	10	R^2^	0.9156	0.8693	0.8773	0.8832	0.8605	0.8731
			RMSE(g/m^2^)	127.02	170.35	167.32	164.22	175.45	168.50
	Spe+Tex+Col (Co)	13	R^2^	0.9793	0.9331	0.9642	0.8817	0.9633	0.9038
			RMSE(g/m^2^)	87.21	124.14	90.62	165.31	91.99	148.99

**Table 3:** Performance of basic and ensemble learning models for estimating AGB on the validation set.

Sensor	Feature type	Variables num	Metrics	Stacking	KNN	GBDT	RF	ADA	SVR
RGB	Tex	5	R^2^	0.6612	0.6104	0.5852	0.5904	0.5903	0.6368
			RMSE(g/m^2^)	272.82	295.70	307.95	302.67	303.49	285.33
	Col	3	R^2^	0.5307	0.4997	0.4802	0.4988	0.5092	0.4270
			RMSE(g/m^2^)	338.11	344.98	351.51	350.22	347.59	369.10
	Tex+Col	8	R^2^	0.7837	0.7447	0.7591	0.7269	0.7471	0.7402
			RMSE(g/m^2^)	208.88	241.09	230.43	249.24	239.21	243.35
MS	Spe (Non-co)	2	R^2^	0.6903	0.6004	0.6671	0.6777	0.6670	0.6217
			RMSE(g/m^2^)	261.90	300.90	270.66	268.32	270.77	290.36
	Spe (Co)	5	R^2^	0.7322	0.7052	0.7127	0.6126	0.7172	0.6041
			RMSE(g/m^2^)	242.94	254.10	251.35	291.73	249.32	294.87
RGB+MS	Spe+Tex+Col (Non-co)	10	R^2^	0.8645	0.8122	0.8226	0.8261	0.8257	0.8081
			RMSE(g/m^2^)	167.82	193.77	188.11	187.33	187.42	195.06
	Spe+Tex+Col (Co)	13	R^2^	0.8819	0.8334	0.8595	0.8030	0.8567	0.8539
			RMSE(g/m^2^)	161.06	199.92	175.13	206.65	176.73	178.90

Spe, spectral features; Tex, texture features; Col, color features; Non-co, Non-collinearity; Co, Collinearity.

### 3.5 Assessment of the applicability of the model

**Figure 10:**
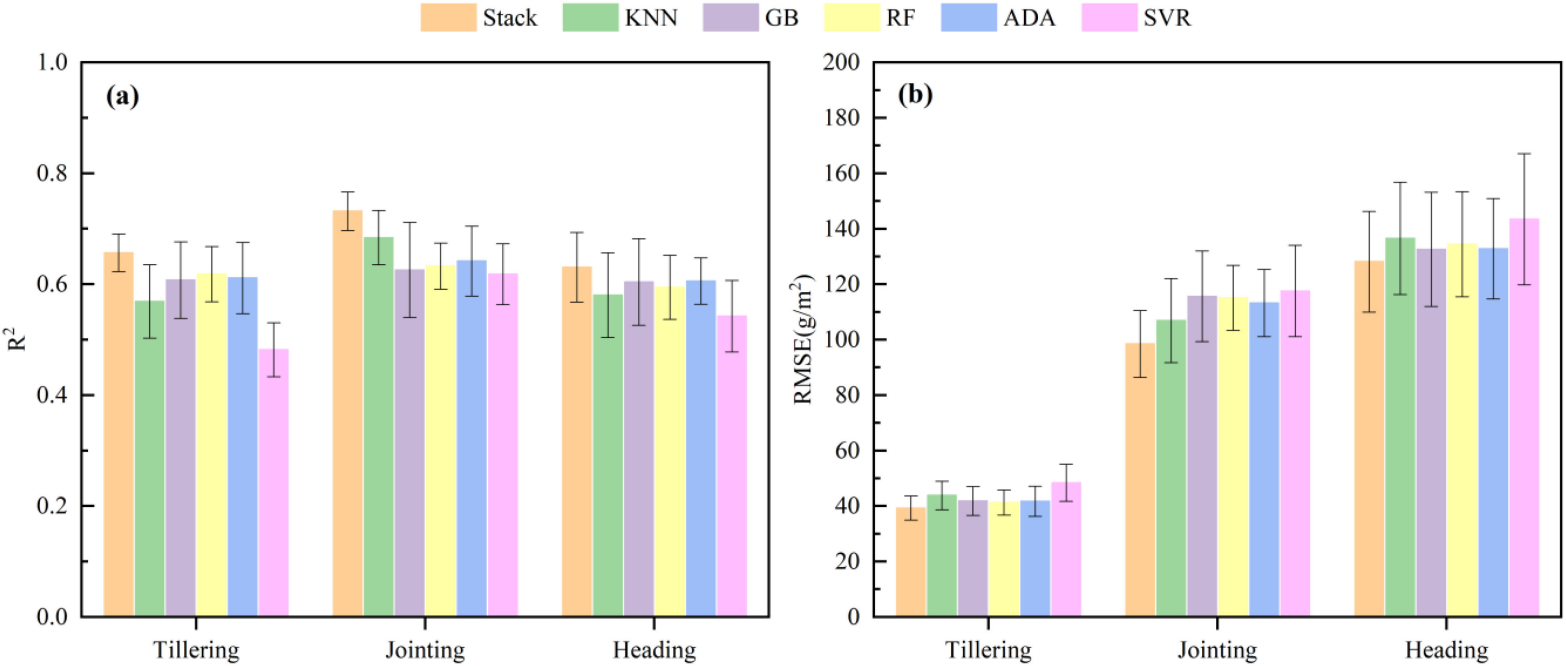
Performance of AGB estimation at different growth periods using the best model of this study. **(a)** Coefficient of determination (R^2^), **(b)** Root mean square error (RMSE).

To further evaluate the applicability and effectiveness of the optimal AGB estimation model developed in this research, the performance of the models was analyzed across three individual rice growth stages, and the results are presented in **Figure 10**. The findings indicate that the Stacking model, which integrates multi-source image feature data, outperforms all base models in terms of AGB estimation accuracy at each growth stage. The average R² values for the model were 0.6509, 0.7565, and 0.6958 at the tillering, jointing, and heading stages, respectively, surpassing the performance of the base models. Additionally, the average RMSE values for the Stacking model were 37.58, 94.86, and 127.05 g/m² at the same stages, respectively, all lower than those of the base models. These results demonstrate the effectiveness of the Stacking model in improving AGB estimation accuracy and stability across different rice growth stages.

## 4 Discussion

### 4.1 Analysis of VIs for estimating rice AGB

**Figure 11:**
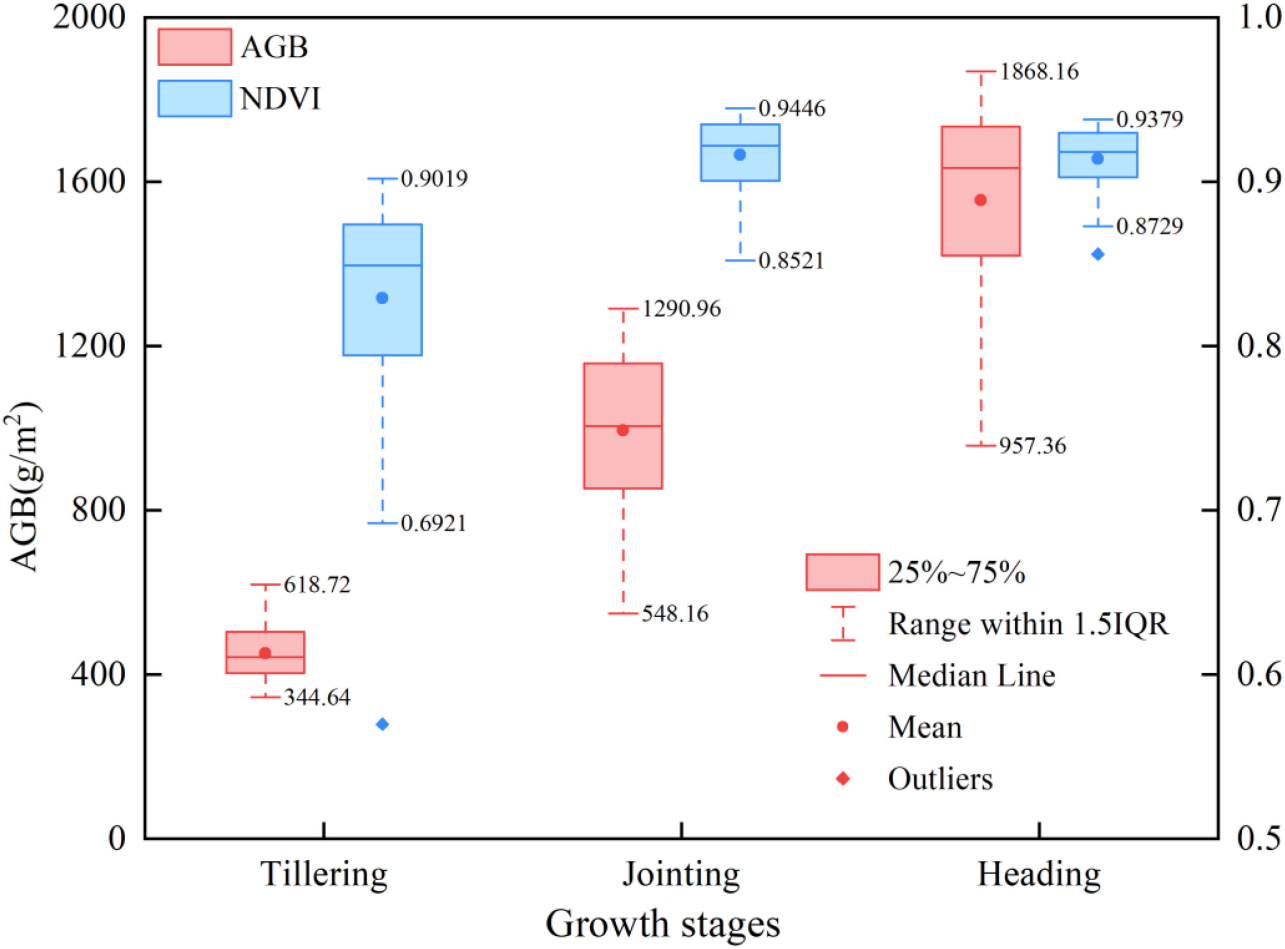
Statistics of changes in NDVI and AGB at different growth periods.

Multispectral-based VIs have been widely used in studies for crop AGB estimation, and their performance varies with band combinations (Cheng et al., 2017; Sun et al., 2023). In this study, it was found that five VIs extracted from MS images were superior for biomass estimation at individual growth stages than when applied across multiple growth stages (**Figure 6**). Among them, two VIs based on red-edge (CIre and MTCI) had the best estimation performance compared to other VIs at the jointing stage (**Figure 6c, e**), and the performance of all VIs decreased significantly at the tillering and heading periods. However, the variation differences in performance among them were small. This was consistent with the conclusions of (Zheng et al., 2019), probably because the red-edge VIs were closely related to the AGB of leaves, but less so to stems or spikes. The canopy was dominated by leaves before the heading stage of rice. However, during the tillering stage, rice plants were relatively small with a sparse canopy structure, and the mixing of the water background in the rice canopy images acquired by the UAV might have affected the VIs calculations, thus reducing the performance of the VIs for estimating AGB. As the rice plants progressed to the jointing stage, leaves became the predominant canopy component, resulting in a closed canopy structure, which minimized the influence of water and other background factors. However, at the heading stage, most plant nutrients were allocated to rice ear development. The canopy was composed of a mixture of stems, leaves, and ears, which also indicated that it was difficult to estimate the biomass of rice ears based on the VIs alone. Furthermore, the AGB increased as the rice grew, but the range of the VIs varied less among the three growth stages. As shown in **Figure 11**, the changes in NDVI from tillering to heading were similar, especially from jointing to heading, but the AGB was increasing rapidly. The present results indicate that estimating rice AGB at multiple growth stages using VIs alone may be limited., which is also consistent with the results in the previous statistical analyses.

### 4.2 Analysis of RGB image features for estimating rice AGB

**Figure 12:**
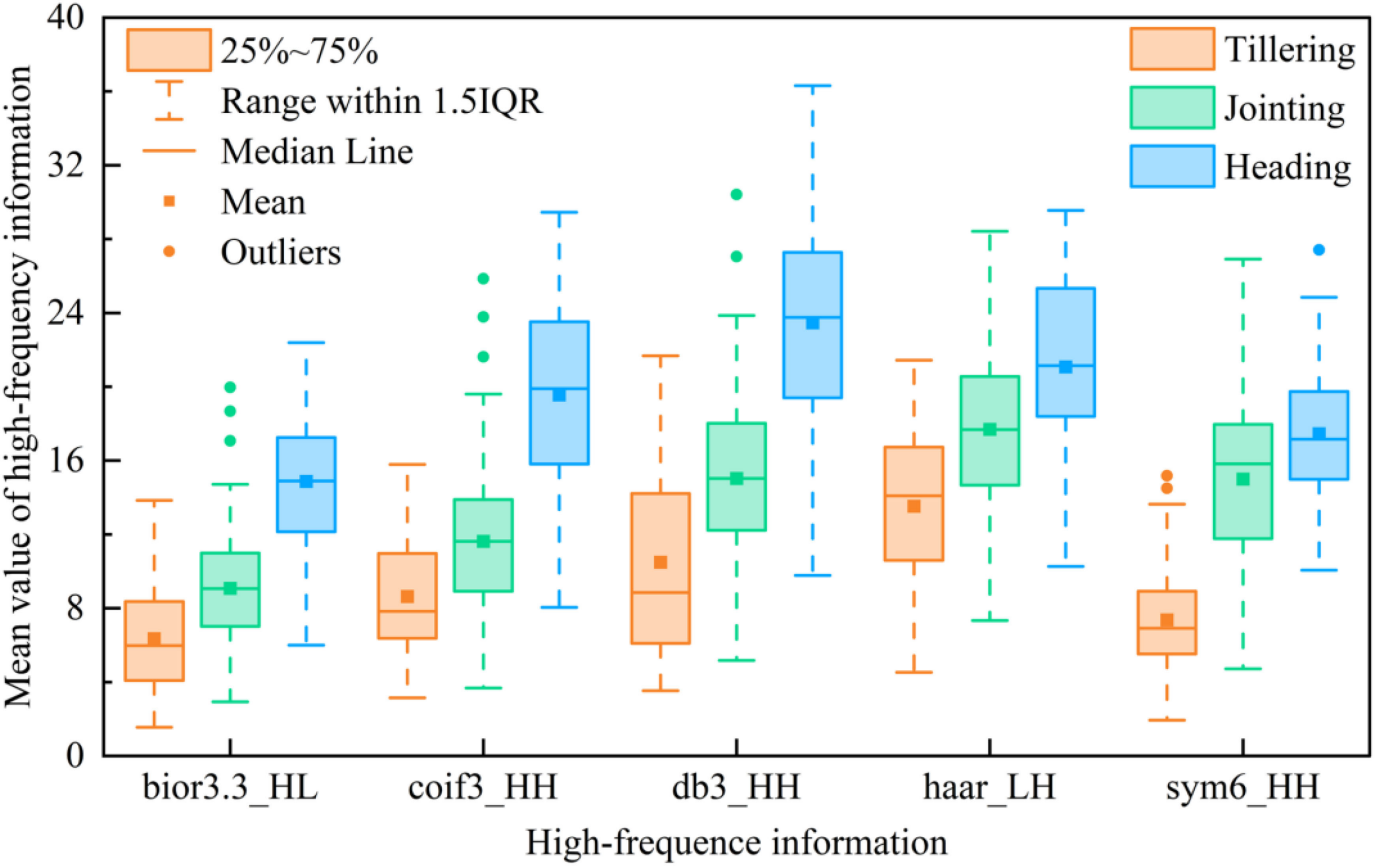
Statistics of changes in high-frequency texture at different growth periods.

**Figure 13:**
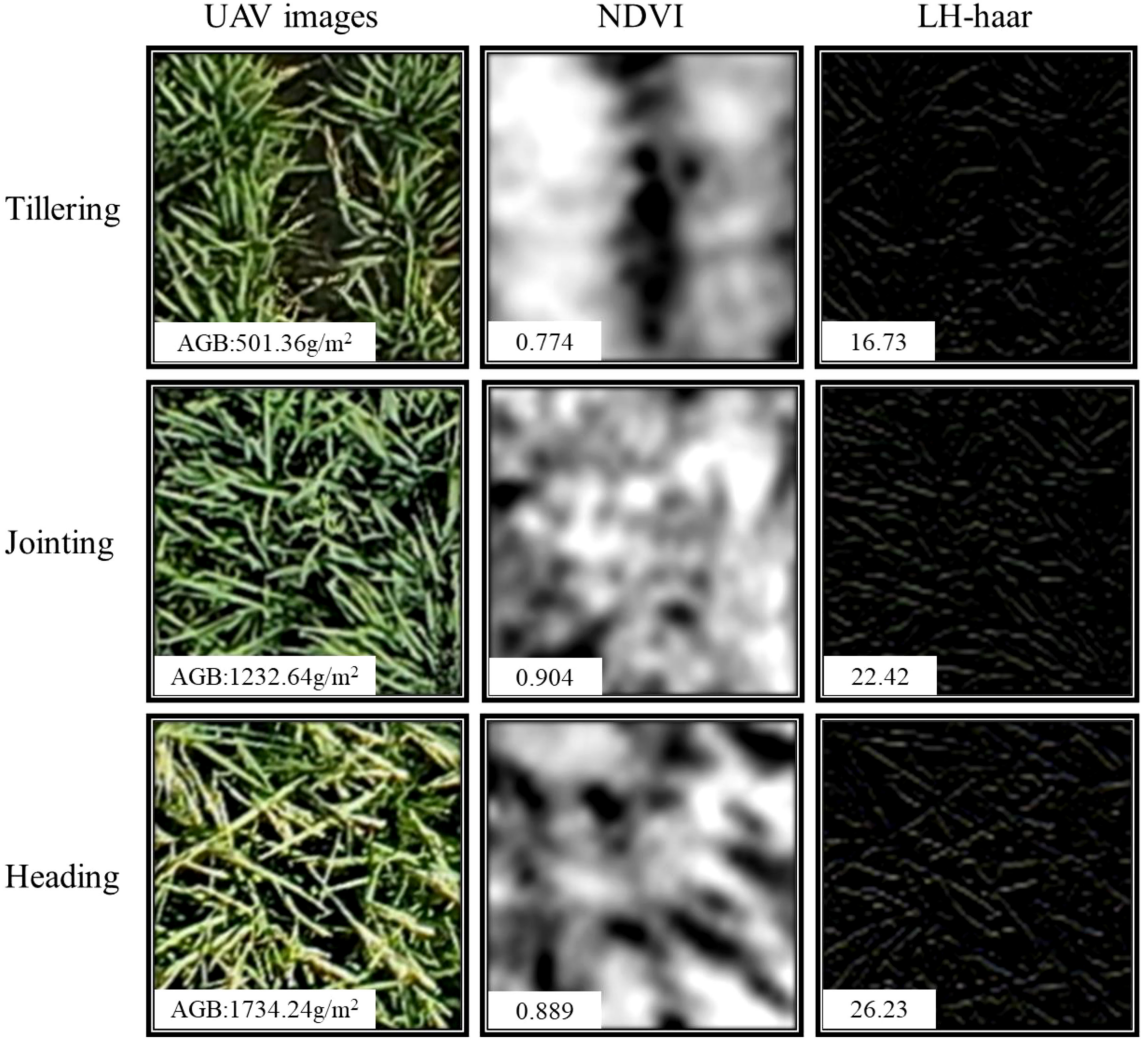
UAV images at different growth periods. NDVI images, and high-frequency texture images.

The spatial and color features of images can increase the data dimension of UAV images with a limited number of bands. Making full use of these features may provide effective technical support for precision agriculture based on UAV images. The rice canopy primarily consisted of stems, leaves, and ears. Consequently, the RGB images of the rice canopy acquired by UAV contained rich high-frequency texture and color information. Compared to MS images, RGB images offer higher resolution and can capture information such as finer color variations and richer texture details. In this study, we used DWT to extract high-frequency texture from rice canopy images in frequency domain analysis. The results showed that the high-frequency texture of the rice canopy image changed significantly with the growth stage of rice (**Figure 12**). The mean value of the optimal high-frequency texture extracted using various small basis functions continuously increased from the tillering to the jointing and heading stage. This is attributed to the canopy structure becoming more complex with the growth of rice. As shown in **Figure 13**, the canopy coverage of rice at the tillering stage was low, resulting in a small extracted high-frequency texture. At the jointing stage, rice leaves shade each other, leading to a relatively closed canopy structure and higher canopy coverage. The emergence of rice ears during the transition from the jointing to the heading stage further enhanced the complexity of the canopy structure. As a result, the high-frequency texture varied with the canopy structure and increased with the increase of AGB. However, the value of NDVI remained constant. The results of the quantitative analysis displayed in **Figure 7**, indicate that high-frequency texture derived from images of rice canopy had the potential to assist in estimating AGB of rice at various growth stages. Similarly (Liu et al., 2023b), found that the application of DWT to extract high-frequency textures from potato canopy images proved to be effective in estimating the AGB of potatoes over multiple growth stages. In addition, we found that the difference in the R^2^ variation of the optimal high-frequency texture extracted through various wavelet basis functions for estimating rice AGB was relatively small (**Figure 7**). This observation suggests that the selection of wavelet basis functions may have a relatively minor impact on the estimation of AGB in rice. We attribute this result to the consistency of different wavelet basis functions in the image decomposition process. Because the wavelet transform achieves feature extraction by decomposing the high-frequency and low-frequency components of the signal, i.e., the basic principles and steps of different wavelet basis functions are consistent in image decomposition. Although different wavelet basis functions vary in mathematical form, they show consistent ability in extracting high-frequency detailed features from rice canopy images. This consistency suggests that in rice biomass estimation, the extraction of high-frequency information mainly relies on common features rather than specific wavelet basis functions, and thus high-frequency information extracted by different wavelet basis functions usually shows similarity in estimation results (**Figure 7**).

**Figure 14:**
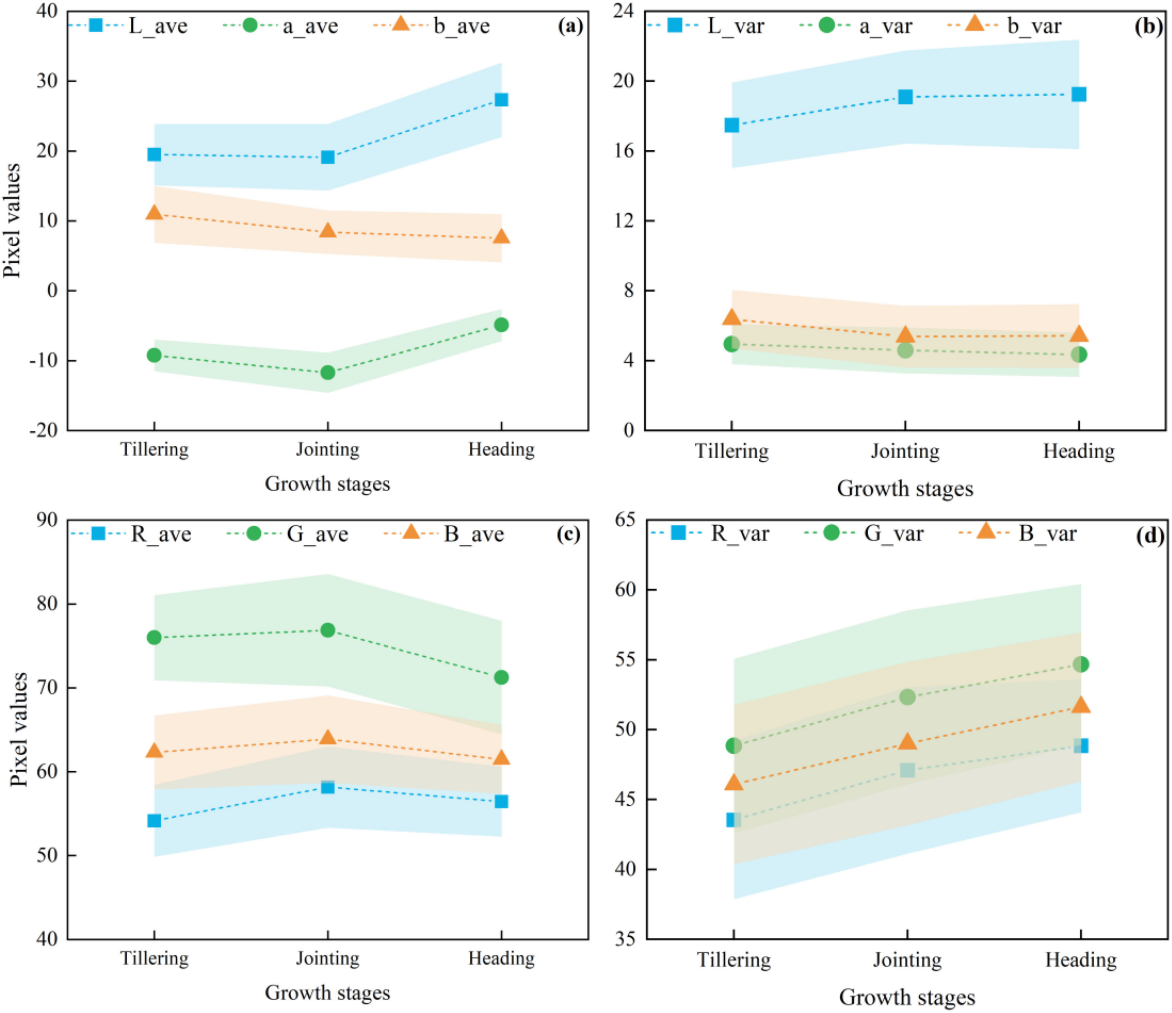
Variation of different color moments at different growth periods. **(a, c)** First-order color moments of Lab and RGB components; **(b, d)** Second-order color moments of Lab and RGB components.

As another important feature of images, color information also plays an important role in multiple growth stages of rice. Color changes at different growth stages can reflect the growth status and developmental process of rice. In studies considering color information as a feature variable, e.g. R, G, B or hue (H), saturation (S), and brightness (V) are often utilized as color features. Related studies such as (Ge et al., 2021b) improved the detection accuracy of the rice growth stage by using the DN values of each color component of RGB and HSV and combining them with the texture features of the images and VIs. However, studies focusing on the color moments calculated in this study to estimate phenotypic parameters such as AGB are rarely reported. In this study, based on the changing law of plant color from green to yellow during rice growth, and combined with a related study (Yang et al., 2024), it is hypothesized that the color information of the image also changes with the growth of rice and the increase of AGB and that by calculating the low-order color moments may be able to further capture the color features in the image effectively. Specifically, rice appeared predominantly green before heading, with the jointing stage exhibiting a darker hue compared to the tillering stage. However, after the heading stage of rice, although the color of the leaves remained green, the rice ears started to appear yellowish-green or golden (UAV images in **Figure 13**). Quantitative analysis, illustrated in **Figure 14**, revealed that the different color moments varied significantly across multiple growth periods. For example, from the tillering to the heading stage, G_ave showed an increase and then a decrease, while a_ave showed a decrease and then an increase. The main reason for this is that the a-component in Lab color space represents the component of color on the red-green axis, and its value is negative the smaller it is, the more green information there is so that the value of a_ave reaches the minimum at the jointing stage. These results indicate that the color characteristics of rice canopy images are closely related to the growth changes of rice. The significant changes in color moments between different growth stages can indirectly and effectively reflect the changes in AGB, providing a strong analytical basis for monitoring rice growth based on image color information.

### 4.3 Comparison of fusion of multi-source image features for estimating rice AGB

To improve the accuracy of estimating AGB over multiple growth periods of rice, and evaluated the ability to fuse feature data from multi-source images for AGB estimation. We explored the fusion and comparative analysis of multi-type feature data extracted from RGB and MS images (**Table 2**). Our study demonstrated that feature data fusion of multi-source images captured by UAV enabled a more accurate estimation of rice AGB over multiple growth periods. Generally, for most ML models, the accuracy of AGB estimation was higher when using feature data fusion from single-sensor or multi-sensor data compared to single-feature data, which was consistent with the results of other studies (Yue et al., 2019). This was because the fusion of feature data such as spectra, texture, and color can offer multidimensional, complementary information, thereby reducing the uncertainty associated with single feature data. In contrast to using expensive UAV hyperspectral data for estimating crop AGB (Liu et al., 2022), integrating various types of features extracted from both digital and MS images in our study also achieved better accuracy in estimating rice AGB and saved the cost of data collection.

### 4.4 The potential of ensemble learning to estimate rice AGB

The fusion of multi-source image feature data provides a more accurate representation of crop growth status. However, analyzing such multi-source and multi-dimensional data presents a substantial challenge. Compared with the traditional linear regression methods, ML techniques are capable of self-learning, self-adaptive, and achieving high-precision regression or classification tasks (Tong and Nikoloski, 2021). Previous studies have utilized ML methods and remote sensing data to estimate phenotypic traits such as crop biomass (Zhang et al., 2021) and yield (Shafiee et al., 2021). It has been observed that most studies typically rely on a single ML method to estimate various crop trait parameters. However, a single ML approach may have limitations when dealing with different types of data. This is reflected in this study as the same method will show different performances under different modeling conditions (**Tables 2**, **3**), indicating the lack of generalization ability and stability in estimating AGB using a single ML method. Due to the limited dataset of 270 sets of data from a one-year trial in this study, the outputs of individual ML models may also have large differences compared to a large sample dataset. Therefore, to integrate the advantages of various ML models and improve the stability of the models. In this study, five ML methods were used as basic models to further improve the estimation accuracy of rice AGB with Stacking ensemble learning. The findings indicated that the ensemble learning model demonstrated superior performance compared to individual ML methods under varying modeling conditions (**Table 2**), confirming the reliability of ensemble learning, which was consistent with previous studies (Feng et al., 2020; Ji et al., 2023). In addition, the advantage of ensemble learning was also demonstrated by comparing the performance of the models in estimating AGB at different growth stages (**Figure 10**). However, there were significant differences in the accuracy of the models at different stages, e.g., the ensemble learning model showed high accuracy at the jointing stage and significantly lower accuracy at the tillering and heading stages. On the one hand, most leaves are in the active photosynthesis stage at the jointing stage contributing more to the canopy spectra. This makes the relationship between canopy features extracted from remote sensing images and AGB more stable, and the model can reflect the changes in AGB more accurately. Therefore, the ensemble learning model was able to make full use of the stable canopy features to provide high prediction accuracy during the jointing period. On the other hand, due to the limited amount of sample data in this study, especially at the tillering and tasseling stages, the diversity of the samples may be insufficient, resulting in the model not being able to fully learn the diverse features at these stages. Compared to the jointing stage, the generalization ability of the model at these stages was constrained by the distribution of data samples, thus showing lower accuracy.

### 4.5 Limitations and prospects

This study focused on evaluating the feasibility and performance of fusing RGB and MS remote sensing image features and applying ensemble learning models to estimate rice AGB at different growth stages. The results demonstrated that the fusion of multi-source UAV image features with ensemble learning can significantly improve AGB estimation accuracy. Therefore, the fusion of multi-source remote sensing data is valuable for the accurate estimation of rice multifertility AGB. However, this study emphasizes the importance of combining UAV RGB images with MS images for AGB estimation at multiple growth stages of rice. These conclusions are based on a one-year dataset involving only two varieties and different N treatment conditions. Therefore, future research should consider using rice datasets across years and locations to validate the applicability and stability of mixing variables, and further focus on how to further estimate rice yield based on accurate AGB estimation. Furthermore, thermal infrared (TIR) imaging, which captures temperature variations within the crop canopy, has been widely utilized for monitoring water stress and predicting crop yield (Maimaitijiang et al., 2020). In the future study, we will integrate UAV thermal infrared data to further explore its potential in rice AGB estimation. While ensemble learning methods have proven effective in enhancing AGB estimation accuracy compared to individual ML models, deep learning techniques offer superior data extraction capabilities (Niu et al., 2021). By using raw multi-source UAV images as input for deep learning models, it may be possible to uncover additional useful features. In subsequent studies, we aim to explore the combination of deep learning with ensemble learning to further assess the potential of multi-source UAV image feature fusion for AGB estimation across multiple growth stages.

## 5 Conclusions

This study evaluated the feasibility of using multi-source image features from UAV RGB and MS to estimate rice AGB at multiple growth stages. To improve estimation accuracy, an ensemble learning model combining five widely used ML techniques was developed. The main findings of the research are as follows:

- The non-normality of the data acquired in this study was determined by the Shapiro-Francia test, and the significant differences between the extracted feature data and AGB were assessed by combining the Kruskal-Wallis pre-test and the Dunn post-test to verify the feasibility of the extracted spectral, texture, and color features to be used for estimating AGB;
- The best VIs, high-frequency texture, and color moment features selected based on the synergistic selection of the statistical selecting method (MIC) and the model selecting method (BorutaShap) explained 43-63%, 38-42%, and 8-20% of the spatial variability of rice over multiple growth stages, respectively;
- Among the various modeling approaches, the fusion of multi-source image features for estimating rice AGB at multiple growth stages effectively reduces estimation errors and enhances model accuracy compared to using single-type features. Additionally, the ensemble learning model constructed in this study significantly improves both the accuracy and stability of AGB estimation when compared to individual ML models.
- The optimal accuracy of rice multi-stage AGB estimation was achieved by fusing multi-source image features using the ensemble learning model, and the R^2^ and RMSE of the validation set were 0.8645 and 167.82 g/m^2^, respectively. Compared to the single model, the R^2^ was improved by 6.4%, 5.1%, 4.6%, 4.7%, and 7.0%, and the RMSE was reduced by 13.4%, 10.8%, 10.4%, 10.5%, and 14.0%, respectively. In addition, the model also achieved satisfactory results in a single key growth stage of rice.

The results of the statistical analysis of data and model estimation in this study indicate that ensemble learning based on combining multi-source image features from UAV-based RGB and MS is feasible and has great potential in estimating rice multi-stage AGB, providing a low-cost method for field management and decision making in precision agriculture. To further evaluate the stability of the method, subsequent studies need to test it under more rice varieties and growing environments.

## Data Availability

The original contributions presented in the study are included in the article/**Supplementary Material**. Further inquiries can be directed to the corresponding authors.
